# Locoregional therapy in luminal-like and HER2-enriched patients with de novo stage IV breast cancer

**DOI:** 10.1186/2193-1801-2-589

**Published:** 2013-11-01

**Authors:** Peng-Yu Chen, Skye Hung-Chun Cheng, Chen-Fang Hung, Ben-Long Yu, Chii-Ming Chen

**Affiliations:** Department of Medical Oncology and Hematology, Koo foundation Sun Yat-Sen Cancer Center, Taipei, Taiwan; Department of Radiation Oncology, Koo foundation Sun Yat-Sen Cancer Center, Taipei, Taiwan; Department of Medical Research, Koo foundation Sun Yat-Sen Cancer Center, Taipei, Taiwan; Department of Surgery, Koo foundation Sun Yat-Sen Cancer Center, Taipei, Taiwan

## Abstract

**Background:**

Locoregional therapy is rarely the standard of care for De Novo stage IV breast cancer but usually used for palliation of symptoms. This retrospective study aimed to determine whether surgery or radiation would contribute to survival benefit for this group of patients by examining the survival outcome through the disease molecular subtypes.

**Materials and methods:**

We reviewed 246 patients with de novo stage IV (M1) breast cancer treated at our hospital between 1990 and 2009. Multivariable Cox Analysis was used to evaluate the survival association with subtypes and clinicopathologic factors.

**Results:**

Patients with luminal-like subtype are mostly premonopausal (66.9%, P = 0.0002), with abnormal CA 15–3 level at initial diagnosis (58.7%, P = 0.01), a higher rate of bony metastases (78.5%, P = 0.02), and a lower rate of liver metastases (22.3%, P < 0.0001). Patients with HER2-enriched and triple negative showed higher rate of nuclear grade III, up to 35% and 40%, respectively (P = 0.01). There is no difference in treatment options patient received: systemic chemotherapy up to 82.2 ~ 95% (p = 0.0705), locoregional treatment up to 40.0 ~ 51.2% (P-0.2571). The median overall survival was 23.1 months: luminal-like subtype 39.6 months, HER2-enriched subtype 17.9 months, and triple negative subtype 13.3 months, respectively (P < 0.0001). In multivariate analysis, poor prognostic factors included HER2-enriched (HR 2.2, P < 0.0001) and triple negative subtype (HR 4.3, P < 0.0001), liver metastasis (HR 1.9, P < 0.0001), lung metastasis (HR 1.4, P = 0.0153), and bone metastasis (HR 1.8, P = 0.0007). Subgroup analysis revealed that local treatments (surgery or radiotherapy) to primary/regional tumors achieved better survival in patients with luminal-like (3-year survival 66.4% vs. 34.4%, p = 0.0001) and HER2-enriched (3-year survival 41.6% vs. 8.8%, p = 0.0012) subtypes, but not in triple negative subtype (P = 0.9575).

**Conclusions:**

For better survival outcome, De Novo Stage IV breast cancer patients with luminal-like or HER2-enriched subtype should be offered local treatments when surgery and/or radiotherapy presents an option for proper control of the primary and regional tumors.

## Introduction

A number of retrospective data have shown that subgroups of women with de novo stage IV breast cancer can attain long-term survival when their distant disease is controlled and their primary tumors are treated. Furthermore, although staged the same, women with de novo breast cancer have superior outcome compared with women with relapsed breast cancer – an indication that de novo breast cancer should be considered as a separate entity entirely (Dawood et al. 2010). Although only limited data linking the intrinsic breast tumor subtype to local treatment benefit for de novo stage IV breast cancer is reported, the strong association between breast cancer subtype and clinical outcome have been pointed out in many studies, such as in ‘patients with early stage breast cancer’ (Ali et al. 2000)’, ‘North American women’ (O’Brien et al. 2010). Furthermore, it is a well accepted fact that luminal subtype achieves better prognosis among the breast cancer subtypes.

It is also known that breast cancer is a group of molecularly distinct disorders according to gene-expression profiling (Sotiriou & Pusztai 2009). However, gene-expression profiling is not widespread use in clinical settings up to the present. Most of us still use hormone receptor (HR) status and human epidermal growth factor receptor-2 (HER2) as predictive and therapeutic markers to select specific therapies for patients with breast cancer (De Laurentiis et al. 2005).

Several retrospective studies have been cited on the suggested strong association between locoregional therapy and improvement of metastatic progression-free survival and overall survival (Khodari et al. 2013). However, there is few data to better define subgroups of patients who would benefit from locoregional therapy, and thus the wide variability in treatment choice always exists.

The question we posed to ourselves was: Does locoreginal treatment for de novo stage IV breast cancer patients have different outcome between different subtypes of patients.

The current guideline in our hospital for the locoregional treatment decisions between palliative and curative for the de novo stage IV patients does not include the molecular subtype as diagnostic factor. This study aimed to determine whether significant survival benefit could be added by locoregional therapy (surgical resection and/or radiation) in patients with de novo stage IV breast cancer.

In this retrospective study, we divided the patients by their intrinsic subtypes, i.e., hormone receptor (HR) and human epidermal growth factor receptor-2 (HER2) status. Based on these markers, breast cancer can be divided into three major molecular subtypes: luminal-like (hormone receptor positive, no HER2 over-expression), HER2-enriched (HER2 over-expression), and triple negative (hormone receptor negative & no HER2 over-expression). The prognosis and clinical outcome from locoregional treatments for de novo stage IV metastatic breast cancer was analyzed.

## Materials and methods

Patients with breast cancer treated between 1990 and 2009 in our hospital who met the following criteria were included in this study: (1) de novo stage IV breast cancer at initial diagnosis, (2) invasive carcinoma of the primary breast tumor, (3) pathological confirmation of metastasis to at least one site. Patients with the following conditions were excluded: (1) prior treatment of systemic chemotherapy or anti-hormonal therapy in other hospitals, and/or (2) second malignancy.

All treatment decisions were made based on the Breast Cancer Clinical Practice Guidelines developed in our hospital in 1993 (Cheng et al. 2000), which are revised annually, and in ways similar to the guideline developed by the National Cancer Center Network (NCCN). All clinical information of patients was collected prior to treatment for a comprehensive Breast Cancer Data Base in our hospital with approval from the Institutional Review Board (IRB). Information collected in this data base consists of (1) general data covering patient demographic information, general medical and family history as well as specific, clinical, and treatment history; (2) pathologic report on tumor tissues; (3) chemotherapy information and related complications; (4) radiotherapy information and related complications; (5) follow-up information submitted every 6–12 months after the completion of all treatments or at a tumor relapse; and (6) late complication, if any.

SAS program was used for data input, data management, data quality control, analysis and presentation. For quality data control, our pathologists record pathological information in the Pathology Report when surgical specimens are available. The clinicians audit the charts to ensure that the clinical information entered into the Breast Cancer Data Base was accurate. All data entries are done twice by two independent data processors. On-line logic check is performed upon the first entry of all data. Logic analysis between data forms and within each form is performed regularly (Cheng et al. 2000).

The clinical risk factors, such as age at diagnosis, primary tumor size, axillary lymph node status, nuclear grade (Kronqvist et al. 1998), lymphovascular invasion (LVI) (Schoppmann et al. 2004), hormonal receptor status, human epidermal growth factor-2 (HER2) and treatments were listed as variables for multivariable analysis on the Cox proportional hazards model.

The Cox proportional hazards regression model was fitted to examine the associations between treatment type and survival (by molecular subtype, with vs. without Locoregional therapy). Overall survival rate of each subtype was calculated through the Kaplan and Meier Method. Log-rank test and Chi-squared test were used to assess the statistical significance.

## Results

### Follow-up and overall survival

A total of 246 patients with de novo stage IV breast cancer with invasive carcinoma treated between 1990 and 2009 were included in this study with the last follow-up date of December 31st 2011. The median follow-up interval was 21 months, and 32 months for 51 patients who were alive. The median overall survival for all 246 patients was 23.1 months; the median overall survival for patients with luminal-like, HER2-enriched, and triple negative subtype were 39.6, 17.9, and 13.3 months, respectively (P < 0.0001).

**Figure 1:**
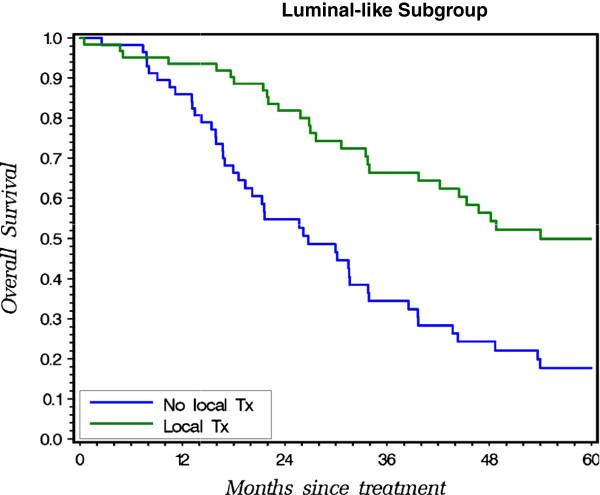
**Probability of OS for patients with vs. without local treatment on Kaplan-Meier Method in Luminal-like Subgroup.**

**Figure 2:**
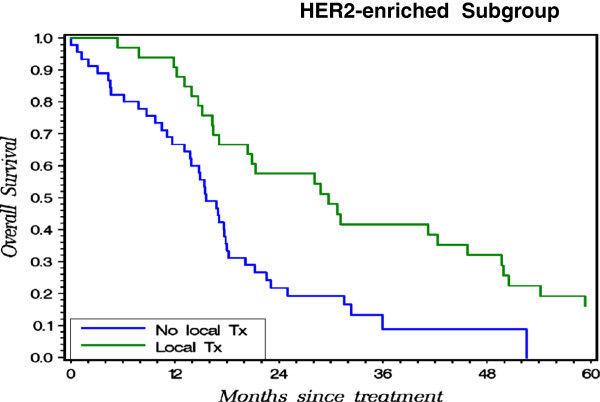
**Probability of OS for patients with vs. without local treatment on Kaplan-Meier Method in HER2-enriched Subgroup.**

**Figure 3:**
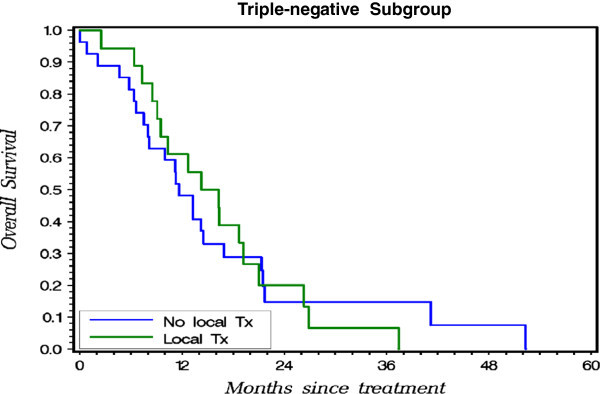
**Probability of OS for patients with vs. without local treatment on Kaplan-Meier Method in Triple-negative Subgroup.**

On the subgroup analysis, locoregional treatment for primary breast tumor showed significant survival benefit in patients with luminal-like (Figure 1, P = 0.0001) and HER2-enriched (Figure 2, P = 0.0012) subtype. With local treatment, the luminal-like patients achieved a 3-year overall survival rate of 66.4% vs. 34.4% for those without the local treatment (Figure 1); for the HER2 rich patients - 41.6% with vs. 8.8% without the local treatment (Figure 2); and for the triple negative 6.7% with vs. 14.8% without. In triple negative subtype, local treatment did not improve outcome (Figure 3, P = 0.9575).

Special mentioning of the anti-HER2 treatment therapy is needed here regarding the result from Figure 2. In the twenty years between 1990 and 2009, the systemic chemotherapies for breast cancer have evolved more than once, especially for HER2-directed therapies. Total number of our patients who received anti-HER2 treatment is too small to ascertain whether locoregional treatment still has significant benefit after those who received anti-HER2 treatment. Nowadays, HER2-directed therapy is widely offered, so for this particular subgroup of patients who get better controlled of systemic disease, the benefit of locoregional treatment may need further investigation.

### Clinical and pathological characters by subtype

Table 1 compares the clinical and pathological factors in each subtype or, (compares the patient characteristics stratified by the three subgroups). The majority of patients in all three subgroups were older than 40 years old. Among the luminal-like patients, 66.9% were premenopausal, which was significantly higher than the HER2-enriched or triple negative subtypes. Direct tumor invasion to chest wall from primary tumor or inflammatory breast cancer (T4 and non-T4 disease) did not stand out in any particular subtype group. Patients with HER2-enriched or triple negative subtype had a higher rate of nuclear grade III of primary breast tumor, up to 35% and 40% (p = 0.0092), respectively. The percentages of prominent lymphovascular invasion of primary breast tumor shown in the subtypes were close. The same similarity was seen in the percentage of locoregional lymph node involvement. Patients with luminal-like subtype had a higher elevation rate of CA 15–3 (58.7%, p = 0.0063).

**Table 1:** **Patient and tumor characteristics by molecular subtype**

	Luminal-like	HER2 -rich	Triple negative	p
	(n = 121, 49.2%)	(n = 80 , 32.5%)	(n = 45, 18.3%)	
**Age**				
> 40	94 (77.7%)	67(83.8%)	36 (80%)	0.5739
≤ 40	27 (22.3%)	13(16.2%)	9 (20%)	
**Menopause status**				
Premenopausal	81 (66.9%)	30(37.5%)	22 (48.9%)	0.0002
Postmenopausal	40(33.1%)	50(62.5%)	23(51.1%)	
**Primary tumor size**				
cT1-3	56(46.3%)	36(45.0%)	23(51.1%)	0.7974
cT4	65(53.7%)	44(55.0%)	22(48.9%)	
**Nuclear grade**				
Nuclear Grade 1-2	24(19.8%)	9(11.3%)	1(2.2%)	0.0092*
Nuclear Grade 3	35 (28.9%)	28(35.0%)	18 (40.0%)	
unknown	62(51.3%)	43(53.7%)	26(57.8%)	
**Lymphovascular invasion**				
No / Focal	15(12.4%)	14(17.5%)	5(11.1%)	0.22121*
prominent	30(24.8%)	12(15.0%)	9(20.0%)	
unknown	76(62.8%)	54(67.5%)	31(68.9%)	
**Lymph node status**				
No axillary LN involvement	30 (24.8%)	19(23.8%)	8 (17.8%)	0.6284
Axillary, IM, or SC LN involvement	91(75.2%)	61(76.2%)	37(82.2%)	
**Tumor markers**				
Elevated CEA	53 (43.8%)	40(50.0%)	15 (33.3%)	0.2740*
Elevated CA 15-3	71 (58.7%)	34(42.5%)	18 (40.0%)	0.0063
**Metastasis site at first diagnosis**				
Liver	27 (22.3%)	43(53.8%)	18 (40%)	< 0.0001
Lung	42 (34.7%)	34(42.5%)	18 (40%)	0.5189
Brain	15(12.4%)	13(16.3%)	8(17.8%)	0.6041
Bone	95 (78.5%)	53(66.3%)	26 (57.8%)	0.0187
**Treatments**				
Systemic chemotherapy	106 (87.6%)	76(95.0%)	37 (82.2%)	0.0705
Anti-hormonal treatment	24 (19.8%)	6(7.5%)	1 (2.2%)	0.0024
Local treatment to primary breast tumor	62(51.2%)	33(41.3%)	18(40.0%)	0.2571

*About whom information was known.

Luminal-like subtype patients had a lower rate of liver metastasis (22.3%, p < 0.0001) and higher rate of bone metastasis (78.1%, p = 0.0187). No significant difference was seen in lung or brain metastasis among the subtype groups.

About the treatment options, more than eighty percent of patients in each group underwent systemic chemotherapy and up to 40 to 50 percent of patients underwent locoregional treatment of primary tumor (p > 0.05). There is no difference in treatment options patient received among these three groups.

### Univariate and multivariate analysis

Table 2 shows the univariate and multivariate analysis of each clinicopathologic factors associated with overall survival. Both the univariate and multivariate analysis showed that HER2-enriched and triple negative subtypes were significantly associated with poor survival. Compared with the luminal subtype patients, the HER2-enriched and triple negative patients had poorer survival on univariate analysis (HER2-enriched: HR 2.0, P < 0.0001; triple negative: HR 4.2, P < 0.0001) and also on multivariate analysis (HER2-enriched: HR 2.2, P < 0.0001; triple negative: HR 4.3, P < 0.0001). This result is consistent with the conclusions illustrated on the Kaplan-Meier curves (Figures 1,2 &3).

**Table 2:** **Overall survival estimates in de novo stage IV patients on univariate and multivariate models**

	Univariate			Multivariate		
Variable	HR	95%CI	P	HR	95% CI	P
**Luminal-like**	1			1		
**HER2–enriched**	2.0	1.4-2.8	< 0.0001	2.2	1.5-3.1	< 0.0001
**Triple negative**	4.2	2.8-6.2	< 0.0001	4.3	2.9-6.5	< 0.0001
**cT1-3 vs. cT4**	0.7	0.5-0.9	0.011			
**NG 1–2 vs. NG 3**	0.9	0.5-1.4	0.5386			
**LVI**						
**focal vs. no**	0.6	0.3-1.4	0.2649			
**Prominent vs. no**	0.5	0.3-1.0	0.0616			
**Lymph node involvement**	1.1	0.8-1.5	0.6471			
**CEA elevated vs. normal**	1.1	0.8-1.5	0.3969			
**CA 153 elevated vs. normal**	1.1	0.8-1.4	0.6379			
**Metastasis**						
**Liver, yes vs. no**	2.3	1.7-3.2	< 0.0001	1.9	1.4-2.5	0.0001
**Lung, yes vs. no**	1.7	1.3-2.2	0.0004	1.4	1.1-1.9	0.0153
**Brain, yes vs. no**	1.5	1.1-2.3	0.0256	1.3	0.9-1.9	0.1728
**Bone, yes vs. no**	1.3	0.9-1.8	0.1016	1.8	1.3-2.5	0.0007
*Local treatment, yes vs. no	0.5	0.4-0.6	< 0.0001	0.6	0.4-0.8	0.0008

Note: *Local treatment: surgery, radiotherapy, or both.

Association between survival and factors such as the clinical staging, locoregional lymph node involvement, nuclear grade, status of lymphovascular involvement of primary tumor, and level of initial tumor markers was not seen in this study. Visceral organ metastasis, including liver, lung, and brain, was an independent factor significantly associated with worse survival on univariate analysis. On multivariate analysis, visceral metastasis also showed a trend to worse survival. Bone metastasis was not significantly associated with survival on univariate analysis but it was an independent prognostic factor on multivariate analysis.

Patients undergoing locoregional treatment to primary breast tumor, including surgical resection and/or radiation, had better survival (HR 0.5, p < 0.0001). The status of visceral organ metastasis may also be one of the deciding factors when considering the locoregional therapy.

## Discussion

Based on our results, it seems reasonable to further stratify the de novo stage IV breast cancer patients into groups of different risk levels by hormone and HER2 status. Our data suggests the breast cancer subtype should be included in decision criteria of local treatment for patients with de novo stage IV breast cancer.

In this study, clinicopathologic factors, such as clinical T stage, nuclear grade, status of lymphovascular infiltration, regional lymph node involvement, and level of tumor markers, did not have impact on the treatment outcome significantly. Most of these clinicopathologic factors were known to associate with recurrence or prediction of treatment response for early stage breast cancer (Corben 2013). For patients with de novo stage IV status, these clinicopathologic factors may play a limited role in managing the outcome.

Some limitations expected of most retrospective type of study are noted here, such as selection bias. The observation presented here is based on the experience of a single institution which may reflect the clinical routine practice of our hospital only.

In summary, breast cancer is a group of molecularly distinct neoplastic disorders. A multidisciplinary approach combining systemic therapies with local treatment (surgery or local radiation) in de novo stage IV patients with luminal-like or HER2-enriched subtypes may not only prevent local complications, but also prolong survival.

## Availability and requirements

The availability and requirements for database are from patients data between 1990 to 2009 in Koo Foundation Sun Yat-Sen Cancer Center.

## References

[CR1] Ali A, Provenzano E, Bartlett JM (2000). Prognosis of early breast cancer by immunohistochemistry defined intrinsic sub-types in patients treated with adjuvant chemotherapy in the NEAT/BR9601 trial. Breast Cancer Res Treat.

[CR2] Cheng SH, Tsou MH, Liu MC (2000). Unique features of breast cancer in Taiwan. Breast Cancer Res Treat.

[CR3] Corben AD (2013). Pathology of invasive breast disease. Surg Clin North Am.

[CR4] Dawood S, Broglio K, Ensor J (2010). Survival differences among women with de novo stage IV and relapsed breast cancer. Ann Oncol.

[CR5] De Laurentiis M, Arpino G, Massarelli E (2005). A meta-analysis on the interaction between HER-2 expression and response to endocrine treatment in advanced breast cancer. Clin Cancer Res.

[CR6] Khodari W, Sedrati A, Naisse I (2013). Impact of loco-regional treatment on metastatic breast cancer outcome: a review. Crit Rev Oncol Hematol.

[CR7] Kronqvist P, Kuopio T, Collan Y (1998). Morphometric grading of invasive ductal breast cancer. I. Thresholds for nuclear grade. Br J Cancer.

[CR8] O’Brien KM, Cole SR, Tse CK (2010). Intrinsic breast tumor subtypes, race, and long-term survival in the Carolina breast cancer study. Clin Cancer Res.

[CR9] Schoppmann SF, Bayer G, Aumayr K (2004). Prognostic value of lymphangiogenesis and lymphovascular invasion in invasive breast cancer. Ann Surg.

[CR10] Sotiriou C, Pusztai L (2009). Gene-expression signatures in breast cancer. N Engl J Med.

